# Supplementary material for: Can empathy be taught? A cross-sectional survey assessing training to deliver the diagnosis of end stage renal disease
Source: PLoS One. 2021 Sep 8;16(9):e0249956. doi: 10.1371/journal.pone.0249956 (PMC8425537; doi:10.1371/journal.pone.0249956)
Supplement: S4 File — S1 Item. Satisfaction and impact survey at the end of the session. S2 Item. Long-term impact survey (spring 2019). (DOCX) [file pone.0249956.s004.docx]

## Supporting Information

## S1 Item. Satisfaction and impact survey at the end of the session

1. Did you find the training session useful?
2. On a scale from 1 to 10, how would you grade the usefulness of the training session?
3. Was the duration of the session appropriate?
4. Do you think that the training session will change your medical practice?
5. Do you think the patient testimony movie will change your medical practice?
6. Any comment:

## S2 Item. Long-term impact survey (spring 2019)

1. Had the training session modified your medical practice in the weeks that followed?
2. Does the training session still influence your current medical practice?
3. If yes, could you give us an example?
