# Supplementary material for: Can empathy be taught? A cross-sectional survey assessing training to deliver the diagnosis of end stage renal disease
Source: PLoS One. 2021 Sep 8;16(9):e0249956. doi: 10.1371/journal.pone.0249956 (PMC8425537; doi:10.1371/journal.pone.0249956)
Supplement: Attachment — Submitted filename: Response to reviewers.docx [file pone.0249956.s005.docx]

**Journal Requirements:**

1. Please ensure that your manuscript meets PLOS ONE's style requirements, including those for file naming. The PLOS ONE style templates can be found at

<https://journals.plos.org/plosone/s/file?id=wjVg/PLOSOne_formatting_sample_main_body.pdf> and

[https://journals.plos.org/plosone/s/file?id=ba62/PLOSOne_formatting_sample_title_authors_affiliations.pdf](about:blank)

We rechecked and confirm our manuscript meets PLOS ONE’s style requirements (title, main body, references and authors affiliations)

2. Please include additional information regarding the survey or questionnaire used in the study and ensure that you have provided sufficient details that others could replicate the analyses.

For instance, if you developed a questionnaire as part of this study and it is not under a copyright more restrictive than CC-BY, please include a copy, in both the original language and English, as Supporting Information.

The Jefferson Scale of empathy used in the study seems to be under copyright. Hence we unfortunately cannot provide a copy in Supporting Information.

*“Copyright ©2001 by the authors. Health Policy Newsletter is a quarterly publication of Thomas Jefferson University, Jefferson Health System and the Office of Health Policy and Clinical Outcomes, 1015 Walnut Street, Suite 115, Philadelphia, PA 19107.”*

(https://www.jefferson.edu/academics/colleges-schools-institutes/skmc/research/research-medical-education/jefferson-scale-of-empathy.html)

 3. Please upload a copy of Supporting Information S1 item and S2 item, which you refer to in your text on page 14.

Supporting information S1 item and S2 item were added to the manuscript.

4. Please amend either the abstract on the online submission form (via Edit Submission) or the abstract in the manuscript so that they are identical.

Abstract on the online submission form was amended

5. Please review your reference list to ensure that it is complete and correct. If you have cited papers that have been retracted, please include the rationale for doing so in the manuscript text, or remove these references and replace them with relevant current references. Any changes to the reference list should be mentioned in the rebuttal letter that accompanies your revised manuscript. If you need to cite a retracted article, indicate the article’s retracted status in the References list and also include a citation and full reference for the retraction notice.

We have reviewed our reference list and ensured it is complete and correct, with no duplication or retracted papers.

**Reviewers' comments:**

Reviewer's Responses to Questions

Reviewer #1: This study evaluates an educational intervention for nephrology trainees in France to improve how the diagnosis of kidney failure is communicated with patients. This is a very novel study, and addresses an important problem in kidney disease care. It is well written and provides important information that can be used internationally to better train nephrologists.

I have one suggestion: Throughout the manuscript, it is recommended that the term “End-Stage Kidney Disease”- or better yet, kidney failure- be used instead of ESRD. See for example: Nomenclature for kidney function and disease: executive summary and glossary from a Kidney Disease: Improving Global Outcomes consensus conference - <https://academic.oup.com/ndt/article/35/7/1077/5880573>

 Thanks a lot for this suggestion. We have replaced End Stage Renal Disease and ESRD by kidney failure throughout the manuscript.
